# Nanostructured Gas Sensors: From Air Quality and Environmental Monitoring to Healthcare and Medical Applications

**DOI:** 10.3390/nano11081927

**Published:** 2021-07-26

**Authors:** Xiaohu Chen, Michelle Leishman, Darren Bagnall, Noushin Nasiri

**Affiliations:** 1 NanoTech Laboratory, School of Engineering, Faculty of Science and Engineering, Macquarie University, Sydney, NSW 2109, Australia; jayden.chen@hdr.mq.edu.au; 2 Department of Biological Sciences, Macquarie University, Sydney, NSW 2109, Australia; michelle.leishman@mq.edu.au; 3 School of Engineering, Faculty of Science and Engineering, Macquarie University, Sydney, NSW 2109, Australia; darren.bagnall@mq.edu.au

**Keywords:** air quality, environmental monitoring, breath analysis, nanostructured gas sensors

## Abstract

In the last decades, nanomaterials have emerged as multifunctional building blocks for the development of next generation sensing technologies for a wide range of industrial sectors including the food industry, environment monitoring, public security, and agricultural production. The use of advanced nanosensing technologies, particularly nanostructured metal-oxide gas sensors, is a promising technique for monitoring low concentrations of gases in complex gas mixtures. However, their poor conductivity and lack of selectivity at room temperature are key barriers to their practical implementation in real world applications. Here, we provide a review of the fundamental mechanisms that have been successfully implemented for reducing the operating temperature of nanostructured materials for low and room temperature gas sensing. The latest advances in the design of efficient architecture for the fabrication of highly performing nanostructured gas sensing technologies for environmental and health monitoring is reviewed in detail. This review is concluded by summarizing achievements and standing challenges with the aim to provide directions for future research in the design and development of low and room temperature nanostructured gas sensing technologies.

## 1. Introduction

From smog hanging over cities to smoke inside the home, air pollution poses a major threat to both climate and health. Poisonous gases, such as NO_2_, SO_2_, and CO_2_ etc. acidic gases and basic gas of NH_3_, in the environment are posing increasingly risks to the ecosystem and public health due to the intensification of human activity. For instance, emitted SO_2_ gas oxidation to SO_3_ followed by reaction with water droplets (moisture) leads to the formation of acid rain (H_2_SO_4_), which causes skin irritation and ulcers, decreases soil fertility, corrodes metallic objects and increases the acidity of water bodies [1,2]. In addition, CO_2_ and NO_2_ gas molecules can readily dissolve in the water droplets in clouds, causing the formation of H_2_CO_3_ and HNO_3_, respectively, affecting natural balance of rivers, lakes and soils and damaging wildlife and vegetation [2,3]. 

Among gases that cause the most air pollution, volatile organic compounds (VOCs) emissions are amongst the biggest environmental problems today [4]. When exposed to sunlight, VOCs react with other gases to form ground-level smog compounds, which stimulate diseases in plants, inhibit seed production and hinder fertilization [5]. Prolonged exposure to VOCs can cause severe health issues in humans including chronic obstructive pulmonary disease, lung cancer and strokes [6]. According to the World Health Organization (WHO), the combined effects of indoor and outdoor air pollution cause about 7 million premature deaths every year [7], and it is estimated that this number will double by 2050 [8]. 

In addition to air pollution, several VOCs present in the human breath are related to important metabolic processes and if detected can serve as an effective non-invasive tool for health monitoring [9]. The concentration of endogenous compounds, including inorganic gases (e.g., nitric oxide, carbon monoxide) and VOCs (e.g., ethane, pentane, ammonia, acetone and ethanol), can be altered in the breath of patients with specific pathologies and, thus, can be utilized as breath markers for diseases [10,11]. While conventional gas sensing techniques such as gas chromatography–mass spectrometry (GC–MS) are gold standard, they are expensive and time-consuming, greatly limiting the potential for real-time measurement. In the past decade, efforts have been shifting from traditional chemical and imaging diagnostic methods to the biotechnology and commercial electronic industries for early-stage and point-of-care diagnostics [12]. A paradigm shift may be offered by the convergence of novel nanoelectronic technologies and big data analytical methodologies [13], providing novel opportunities to improve the quality of healthcare while decreasing costs by the very early-stage detection and prevention of fatal and chronic diseases [9,10,12,13].

The use of advanced nanostructured sensing materials is one of the promising detection techniques for monitoring low concentrations of VOCs in a complex gas mixture, for air quality, environmental monitoring [14], health, and medical applications [15]. Among many nanostructured gas sensing technologies, metal oxide semiconductors represent a class of unique materials due to their low cost, high sensitivity, simple fabrication and excellent reproducibility [9]. However, high operating temperature and consequently, excessive power consumption and poor long-term stability, are significant operational challenges [9], hindering their real-world application as miniaturized portable gas sensors [10]. There are continuing efforts toward the development of efficient sensing technologies for room temperature operation devices either by tailoring the property of the sensing layer with desired morphologies or designing novel nanostructures exploiting distinct properties of some nanomaterials.

Here, we present some of the key innovations in nanostructured sensors that are leading the way in sensitive and selective gas sensing at low operating temperatures. We focus on the impact of the nanoscale material hierarchy, discussing the similarities and differences across nanomaterials, the nanostructured morphologies as well as sensing mechanisms for low temperature detection of VOC gases in different sensing environments. We conclude with a review of issues that need to be overcome to enable the engineering of the next generation of miniaturized low-temperature nanostructured gas sensors for air quality, environmental monitoring, health, and medical applications. 

## 2. Air Quality and Environmental Monitoring

With the increasing trend towards urbanization, concern for high levels of pollution and greenhouse emissions due to higher demands on transport, and energy consumption is increasing day by day [16]. Air pollutants and greenhouse gases, especially high levels of nitrogen dioxide (NO_2_), sulphur dioxide (SO_2_) and carbon dioxide (CO_2_) are considered as the most significant environmental risks to public health in urban areas as well as climate change around the globe [17].

### 2.1. Nitrogen Dioxide (NO_2_)

NO_2_ is a highly poisonous toxic gas produced as a byproduct of internal combustion engines in vehicles, and the household or commercial combustion of coal, gas, oil, or wood [18,19]. It is one of the most common air pollutants (particulate matter, ozone, NO_2_ and SO_2_) that is used as an air quality indicator globally by the World Health Organization (WHO) [20]. NO_2_ is considered dangerous for human health at concentrations greater than 4 parts per million (ppm) at which it can anesthetize our sense of smell [1], and from there overexposure results in respiratory and cardiovascular illnesses and diseases [19]. NO_2_ can cause severe environmental problems such as smog [21], acid rain [22,23], and nutrient pollution in coastal waters [24]. According to the U.S. Occupational Safety and Health Administration (OSHA), the permissible exposure limit for toxic industrial NO_2_ gas is 5 ppm [25]. Therefore, the development of sensitive and selective NO_2_ gas sensors with low ppm detection and operating without heating requirements is critical for many practical applications, including continuous monitoring of air quality and health. 

Among a wide range of sensing materials, metal oxide semiconductors have attracted significant attention due to their low cost, easy fabrication, simplicity of use, large number of detectable gases, compact size and simple sensing operation [9,15]. However, their high operating temperature (to accelerate the sensing kinetics) and consequently high-power consumption, hinder their real-world application as portable and/or wearable devices [10]. In addition, they suffer from long-term stability as their high operating temperature induces nanostructure grain growth that can result in the significant reduction of sensing performance [9]. The urgent task, therefore, is to reduce the operating temperature of metal oxide-based gas sensors.

**Figure 1:**
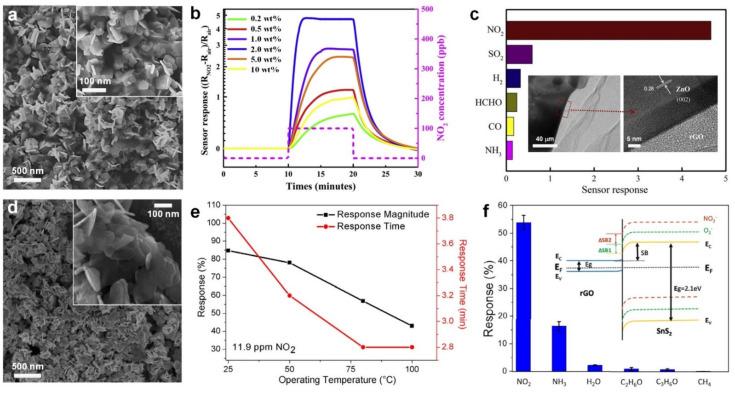
(**a**) FE-SEM images and (**b**) the sensing performance of rGO/ZnO_1−x_ nanocomposite towards NO_2_ gas with 100 ppb concentration. (**c**) Sensor’s selectivity towards NO_2_ (100 ppb) in comparison with SO_2_ (100 ppm), H_2_ (400 ppm), HCHO (10 ppm), CO (100 ppm), NH_3_ (100 ppm) under white light illumination at room temperature. Reproduced with permission from [26] Elsevier, 2019. (**d**) SEM images and (**e**) sensing response of hybrid SnS_2_-rGO sensor in the presence of 11.9 ppm NO_2_ as a function of operating temperatures. (**f**) Sensor selectivity towards NO_2_ (11.9 ppm), NH_3_ (99 ppm), ethanol (50 ppm), acetone (50.5 ppm), CH_4_ (10 ppm) at 50% relative humidity (RH) and 80 °C. Reproduced with permission from [30] Elsevier, 2018. Inset: band realignment and energy diagram of SnS_2_/rGO sensors.

For this purpose, Geng et al. [26] reported the utilization of white-light illumination, as an external activation energy source, to accelerate the sensing response (also termed as gas sensitivity, defined as $S=\frac{\left|R_{g}-R_{a}\right|}{R_{a}}$, where $R_{g}$ and $R_{a}$ are the electrical resistance upon exposure to targeted gas and air, respectively) at room temperature. Using a one-step hydrothermal synthesis technique, they developed a p–n heterojunction of reduced graphene oxide (rGO) and ZnO_1−x_ nanocomposite made of enwrapped ZnO nanosheets (Figure 1a) and gauze-like thin layers of rGO (Figure 1c, inset) for ppb-level detection of NO_2_ gas molecules at room temperature. Upon white light illumination, the sensing material exhibited an enhanced response and recovery kinetics compared to the dark condition. This is attributed to the significant change in the light absorption spectra of the rGO/ZnO_1−x_ nanocomposite which was red-shifted and extended to the entire visible light region. In the case of pure ZnO film, white light illumination is not efficient as photons with excitation energies greater than 3.3 eV are required to accelerate the sensing response [27,28]. In contrast, the rGO/ZnO_1−x_ nanocomposite can absorb low-energy visible-light photons, resulting in significantly higher sensing response of 4.66 under white-light illumination compared to 0.31 of the pure ZnO nanofilm at the same conditions [26]. In addition, the fabricated nanocomposite demonstrated a faster response dynamic upon exposure to NO_2_ gas molecules with 1.5- and 2.5-min response and recovery time respectively, compared to that over 10- and 20-min, for the pure ZnO nanofilm. The synergistic effects between ZnO nanosheets, as gas reaction units and the rGO nanolayers, as the photosensitizer provide a strong electron-donor and electron-transfer pathway, and enhances the sensing performance of the nanocomposite compared to the pure ZnO sensor [26].

Figure 1b presents the sensing performance of the fabricated nanocomposite with different rGO/ZnO_1−x_ weight ratio for NO_2_ gas molecules with 100 ppb concentration at room temperature (25 °C). The gas sensing response increased from 0.7 of rGO/ZnO_1−x_ film with 0.2 wt% ratio to 4.66 for the film with 2.0 wt% ratio. This is attributed to the participation of more electrons created during adsorption as electrons are transferred from rGO to ZnO [26]. In addition, the large surface density of adsorption sites in the rGO/ZnO_1−x_ nanocomposite resulted in more physiosorbed NO_2_ on the surface. However, further increase in the weight ratio of rGO/ZnO_1−x_ to 10% reduced the sensing performance of the hybrid device to less than 1. This reduction in sensing performance is attributed to the ZnO nanosheets covered by rGO layers, resulting in a significant reduction in gas molecules adsorption on ZnO surface [26]. Increasing the NO_2_ gas concentration from 50 to 400 ppb resulted in a linear enhancement in sensor’s response from 2.5 to 19. The fabricated nanocomposites demonstrated a linear enhancement in sensing performance upon exposure to NO_2_ gas with concentration increasing from 50 to 400 ppb. Furthermore, the sensing response was investigated upon exposure to a variety of targeted gases with different concentrations (Figure 1c), demonstrating a remarkable selectivity towards NO_2_ (S = 4.6) with a neglectable response to other gases including SO_2_ (S < 0.6, 100 ppm), H_2_ (S < 0.5, 400 ppm)_,_ HCHO (S < 0.4, 10 ppm), CO (S < 0.3, 100 ppm) and NH_3_ (S < 0.2, 100 ppm) [26]. This higher sensitivity towards NO_2_ could be attributed to the higher electron transfer between NO_2_ and the surface of fabricated nanocomposites at room temperature compared to other targeted gases. In fact, a higher operating temperature might be required to enhance the sensitivity of the fabricated sensor towards other gases [29].

In another approach, Shafiei et al. [30] developed a low operating temperature hybrid nanostructured gas sensor made of wet-synthesized SnS_2_ nanoflakes (Figure 1d) drop-casted on a rGO film. The synthesized SnS_2_ nanoflakes demonstrated a typical thickness of 6 nm (Figure 1d, inset) corresponding to only 10 monolayers of 2D hexagonal SnS_2_ flakes. The fabricated sensor deposited on the alumina substrates featuring Au-Ti interdigitated electrodes showed a strong sensing response of 0.85 towards 11.9 ppm NO_2_ at a room temperature of 25 °C. However, a long response time of 3.8 min was recorded while the sensor never recovered to the original baseline. Increasing the temperature to 80 °C, however, reduced the response to a magnitude of 0.57 with full recovery to the baseline resistance (Figure 1e). This reduced sensing response at higher operating temperature is attributed to the competition between adsorption and desorption of targeting gas on and from the surface, indicating the dominant effect of surface desorption of NO_2_ gas molecules [30]. Further increases in the operating temperature up to 100 °C resulted in only a negligible decrease in the response magnitude and response time (Figure 1e), while the recovery speed improved slightly. 

In this case, the sensing mechanism is based on an electrostatic field formation at the interface between SnS_2_ and rGO due to the difference between work functions. This electric field results in the transfer of electrons from rGO to the surface of the SnS_2_ nanoflakes (Figure 1f, inset), leading to higher concentrations of electron and hole charges in the SnS_2_ and rGO films, respectively. Upon exposure to NO_2_ gas, an electron on the surface of SnS_2_ nanoflakes is captured by the gas molecules leading to the formation of negatively charged NO_2_^−^ species (Figure 1f, inset), thereby causing a reduction in film resistance [18]. The response magnitude increased from 0.25 to 0.57 with increasing the NO_2_ gas concentration from 0.75 to 7.5 ppm. However, the response magnitude saturated with no further change in film resistance for the NO_2_ gas concentration higher than 7.5 ppm. The gas selectivity of the developed SnS_2_-rGO films were tested towards a variety of target gases including NH_3_ (ammonia), C_2_H_6_O (ethanol), C_3_H_6_O (acetone) and CH_4_ (methane). No detectable response was observed upon exposure to ethanol, acetone or methane (Figure 1f), demonstrating high selectivity of the film towards these gases while a low response of 12% was detected towards relatively high concentrations of NH_3_ (99 ppm) [30]. This selectivity towards NO_2_ could be attributed to the remarkably high molecular-surface binding energy (140 meV) between the sensing material (SnS_2_) and NO_2_ gas molecules, resulting in higher physisorption rate of NO_2_ gas molecules compared to other gases including CH_4_, C_3_H_6_O and C_2_H_6_O [31]. In a similar approach, SnS_2_/rGO nanohybrids were also fabricated by Huang et al. for ppb-level NO_2_ gas detection [18]. By tuning the ratio of rGO and SnS_2_, the sensors exhibited p- and n-type transitions with remarkable 1–5 ppb limit of detection (LOD) and fast response dynamics at room temperature (Table 1). A combination of visible light (λ = 650 nm, 1 mW·cm^−2^) and a bias of 15 V was used to achieve the room temperature sensing performance [18].

### 2.2. Sulphur Dioxide (SO_2_) 

SO_2_ is another air quality indicator listed in Air Quality Guidelines by the WHO [20], with a threshold limit of 5 ppm for human exposure and a long-term exposure limit of only 2 ppm [32,33]. It is a highly toxic gas with a pungent, irritating, and rotten smell. Natural phenomena such as volcanic eruptions, degradation of organic waste matter and hot springs, or human activities including burning of fossil fuels, metallurgical processing of pyrite and sulfide ores, etc., can result in releasing SO_2_ gas molecules into the atmosphere [1,33]. Inhalation of SO_2_ gas can seriously affect human health including irritation in respiratory tracts and eyes, bronchitis, asthma, permanent pulmonary impairment, and lung cancer [19]. Detection of SO_2_ is an important objective, but considering the corrosiveness of SO_2_ gas, developing a sensitive, selective, and reliable SO_2_ gas sensor for real time monitoring of SO_2_ molecules at room temperature is a significant challenge. 

**Figure 2:**
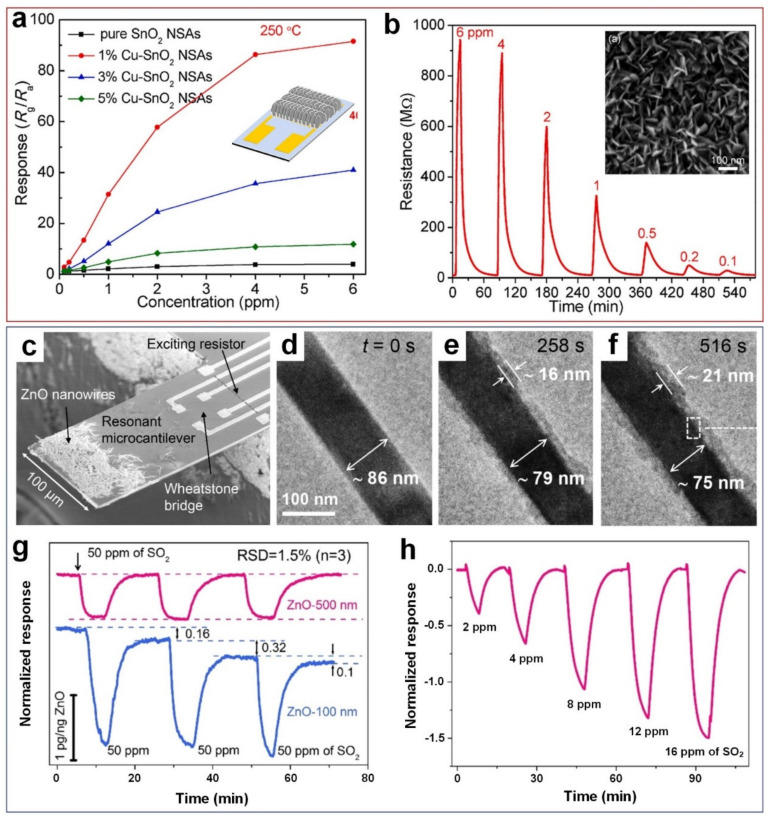
(**a**) Sensing response of Cu-doped SnO_2_ sensors with different Cu content and (**b**) the corresponding dynamic resistance curves of 1% Cu-doped sensors toward SO_2_ with concentrations of 0.1–6 ppm at 250 °C (inset: the SEM image 1%Cu-SnO_2_ sensor). Reproduced with permission from [34] Elsevier, 2020. (**c**) SEM image of on the resonant microcantilever loaded with ZnO nanowires. (**d**‒**f**) In-situ TEM of the ZnO–100 nm sample surface morphology with the presence of SO_2_ gas for different exposure time. (**g**) The sensing dynamics of the ZnO 100 nm and 500 nm samples to SO_2_ gas. (**h**) The sensing performance of ZnO-500 nm sensor to different concentration of SO_2_ gas at 30 °C. Reproduced with permission from [36] American Chemical Society, 2021.

Zhao et al. [34] demonstrated an on-chip growth of ultrathin Cu-doped SnO_2_ nanosheet arrays (Figure 2a, inset) using a homogenous precipitation method that produced vertically aligned flake-like nanosheets (Figure 2b, inset) with an average thickness of less than 10 nm. Using Cu atoms as morphological modifier to generate structural defects, the optimal 1 mol% Cu-SnO_2_ device showed a 90.15 sensing response toward 6 ppm SO_2_ compared to a 2.95 response in the case of pure SnO_2_, at the same 6 ppm SO_2_ concentration (Figure 2a). This higher sensing performance (23-fold) is attributed to the incorporation of Cu dopants into SnO_2_ lattice, the generation of a large concentrations of oxygen vacancies and consequently more adsorption sites in the Cu-SnO_2_ nanostructure [34]. Further increase in the Cu dopant concentration up to 5 mol%, however, reduced the sensor’s response significantly to 10.82 (Figure 2a). This lower sensing performance could be ascribed to the reduction in the available surface area due to the formation of irregular shaped particles within the nanostructure. However, more studies are required to provide a clear understanding on the key role that a particle shape might play in changing the sensing performance of nanostructured devices. In addition, high operating temperature (250 °C) and excessive energy consumption hinders real-world application as portable gas sensors. Optimization of the working temperature is an important functional characteristic as temperature controls the adsorption-desorption equilibrium of the gases at the surface of the sensor, diffusion of the gases, formation and surface density of oxygenated anionic species, and the electronic mobility between the conduction and the valence band of the semiconductor metal oxides [35].

Despite significant progress in developing chemiresistive metal-oxide semiconductor-based gas sensors for SO_2_ gas detection, irreversible reactions between SO_2_ gas molecules and metal oxide sensing materials hinder their room temperature application as a prolonged UV irradiation and heating process is required to recover the sensor to the baseline. Using a solvothermal fabrication technique, Nikesh et al. [33] developed a p-type field effect transistor (FET) based on a nanocomposite made of nickel benzene carboxylic (Ni_3_BTC_2_) and OH-functionalized single-walled carbon nanotube (OH-SWNTs) for reversable detection of SO_2_ gas (4–20 ppm) at room temperature. However, further investigation is required to fully understand the sensing mechanism and the reversibility of such nanocomposites. 

In another study, Wang et al. [36] demonstrated a quantitative analysis of kinetics of solid–gas reactions between ZnO nanowires and SO_2_ gas molecules using in situ TEM technique (Figure 2c) and recorded the real-time morphological evolution of the ZnO nanowires during SO_2_ gas exposure. When the experiment was carried out at room temperature, no obvious morphology change was observed, and the morphology change only occurred when the temperature was increased to 70 °C [36]. As shown in Figure 2d–f, a homogenous shell gradually formed on the outer layer of ZnO-100 nm nanowire due to the diffusion of SO_2_ molecules through the ZnO surface where the shell thickness increased from 0 to 16 nm by increasing the exposure time from 0 to 258 s. This is attributed to the irreversible reaction between SO_2_ gas molecules and ZnO surface where atoms on the nanowires surface were gradually consumed by SO_2_ to form a zinc sulfite hydrate (ZnSO_3_.2.5H_2_O) shell [36]. Further increase in the exposure time up to 516 s led to the formation of a dense shell layer (21 nm) around the ZnO surface (Figure 2f), resulting in the slower reaction rate between SO_2_ gas molecules and atoms on the ZnO surface. 

A different solid–gas reaction kinetic, however, was observed for ZnO nanowires with 500 nm diameter where no obvious morphological change on the smooth surface of the ZnO nanowires over exposure to SO_2_ gas molecules even hours after the reaction was completed. Figure 2g illustrates the sensing response of ZnO-100 nm and ZnO-500 nm nanowires towards 50 ppm of SO_2_ gas molecules at 70 °C. The ZnO-100 nm sample exhibited higher sensing response values than the ZnO-500 nm sample. However, the sensing material never recovered to the baseline (Figure 2g, blue line). In fact, the high reactivity of the ZnO-100 nm sample towards SO_2_ gas is due to the poisoning effect of SO_2_ on ZnO surface, and consequently, irreversible solid-gas reaction between thin nanowires and gas molecules [36]. In contrast, a reversible sensing response to SO_2_ was observed for the ZnO-500 nm nanowires (Figure 2g, pink line), indicating the important role nanostructures play in solid–gas reaction kinetics where ZnO-500 nm is a suitable sample as a reversable SO_2_ sensing material while (Figure 2h) ZnO-100 nm could be used in SO_2_ gas capturing applications. Figure 2h presents the normalized sensing performance of fabricated ZnO-500 nm nanowires towards SO_2_ gas in a concentration range of 2–16 ppm, at low operating temperature of 30 °C. The experimentally observed LOD towards detecting SO_2_ gas molecules was lower than 2 ppm for ZnO-500 nm nanowires. A LOD of 70 ppb was reported using the empirical method and a signal-to-noise ratio of 3 [36]. 

### 2.3. Carbon Dioxide (CO_2_) 

CO_2_ is an odorless, colorless and an important long-lived trace gas that now constitutes up to around 0.04 mol% (415 ppm) of the atmosphere [37]. Despite its relatively small overall concentration, CO_2_ is the major primary driver of climate change among all different types of greenhouse gases [38]. Human emission of CO_2_ has increased significantly since preindustrial times [39], which will potentially cause irreversible damages to the environment in the next coming years [40]. In addition, sustained CO_2_ exposure in indoors settings can cause inflammation, reduction in cognitive abilities and oxidative stress at modest concentration levels (1000 ppm) [41,42]. Many countries have pledged carbon neutrality within the upcoming three decades; the design and fabrication of a stable, reversible, real-time CO_2_ gas sensor with fast response dynamics, ppm level detection and low cost that could be easily deployed in urban cites will greatly assist a pathway towards lower and net-zero CO_2_ emissions [43].

**Figure 3:**
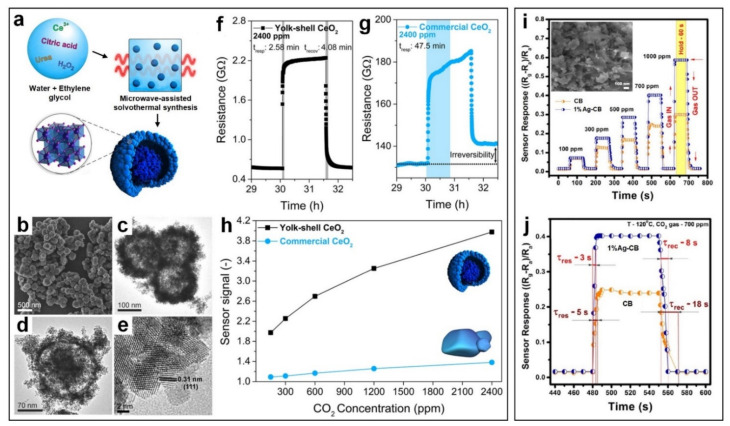
(**a**) Schematic illustration of the microwave-assisted solvothermal synthesis of the yolk-shell CeO_2_ nanospheres. (**b**) FESEM and (**c**–**e**) TEM images of yolk-shell CeO_2_ nanospheres. (**f**,**g**) Response and recovery times of the yolk−shell CeO_2_ and commercial CeO_2_ nanosensors towards 2400 ppm of CO_2_. (**h**) Sensor signal of the yolk-shell and commercial CeO_2_ nanospheres as a function of CO_2_ concentration. Reproduced with permission from [48] American Chemical Society, 2020. (**i**) Response dynamic transients of the Ag@CuO/BaTiO_3_ sensor as a function of CO_2_ concentration at 120 °C (inset: BaTiO_3_ spheroids decorated with CuO microleaves in the equimolar ratio). (**j**) Comparative response and recovery curve of 700 ppm CO_2_ gas at 120 °C. Reproduced with permission from [49] American Chemical Society, 2017.

Current sensing technologies to monitor atmospheric concentration of CO_2_ is based on nondispersive infrared (NDIR) sensors where an IR lamp guides light waves through a sampling tube filled with atmospheric air. The difference between the wavelengths radiated by lamp and the wavelengths absorbed by the detector is used as an indicator for CO_2_ gas concentration. In such sensing systems, an optical filter is placed in front of an IR detector which absorbs all wavelengths generated by the IR lamp, except the ones which are absorbed by CO_2_ molecules. NDIR-based sensors demonstrate acceptable sensitivity and fast response towards a broad CO_2_ concentration range. However, their high-power consumption hinders their utilization in battery-driven devices. Several detection techniques including surface plasmon resonance, surface acoustic wave, fluorescent and colorimetric sensing technologies have been used to develop highly sensitive and selective CO_2_ gas sensors that can operate at ambient temperatures [44]. However, there are still many challenges that restrict the commercial availability of these sensing devices including high complexity of the structural configuration, its large size and high cost [44]. Recently, hollow nanostructures have attracted much attention as promising nano-designed gas sensors due to their unique porous morphology, high specific surface area and effective gas diffusion [45,46,47]. Using a simple microwave-assisted solvothermal method (Figure 3a), Zito et al. [48] developed yolk-shell CeO_2_ nanospheres with an average nanosphere diameter of 190 ± 20 nm (Figure 3b–e), demonstrating an efficient strategy to enhance the sensing performance of metal oxide nanosensors towards CO_2_ gas. At a relatively low temperature (100 °C) and relative humidity of 70%, a high gas response of 2.9 with a fast response and recovery times of 2.58 min and 4.08 min, respectively, was reported for the yolk-shell CeO_2_ nanospheres towards 2400 ppm CO_2_ gas, compared to a sensing response of 0.4 for the commercial CeO_2_ nanoparticles (Figure 3h). This higher sensing performance is attributed to the higher surface area and porosity of the yolk-shell nanosphere, enhancing the permeability to gas adsorption even into the inner part of the nanospheres [47]. Compared to the bulky and impermeable structure of the commercial CeO_2_, this porous nanostructure plays a vital role in providing an effective carrier gas diffusion after CO_2_ pulses, thereby enhancing the recovery speed of the sensing material (Figure 3f,g). This results in a full recovery of yolk-shell nanostructured morphology, compared to partial recovery of commercial CeO_2_, avoiding the formation of any drift in the baseline resistance between gas pulses (Figure 3f,g).

In another approach, a hybrid heterostructure made of tetragonal n-type BaTiO_3_ spheroids decorated with p-type CuO micro-leaves (Figure 3i, inset) was synthesized by a simple hydrothermal method [49], demonstrating an exceptional stability and sensitivity toward CO_2_ gas compared to pure BaTiO_3_ device (Figure 3i). The BaTiO_3_ has a large bandgap of 3.1–3.3 eV with a high carrier concentration of up to 7 × 10^21^ cm^−3^ with minimal lattice mismatch with CuO (<2%), making them promising pairs for the formation of hybrid heterostructure [50,51]. The p–n junction formation results in creating electronic interaction and change in work function of sensing materials, which leads to reduction in the energy barrier at the grain boundary of BaTiO_3_ and CuO [49]. This leads to the formation of a smaller depletion layer and subsequently, higher sensing response upon exposure to CO_2_ gas molecules. The sensing mechanism is based on a reversible reaction between CuO and CO_2_ gas resulting in the formation of copper hydroxycarbonates [49]. Further improvement in sensing response of the fabricated p–n heterojunction material was achieved by incorporation of Ag nanoparticles via wet impregnation. This higher sensing performance could be attributed to the partial oxidization of Ag nanoparticles and the formation of thin Ag_2_O layers which could act as acceptors, facilitating the sensing phenomenon towards CO_2_ gas [49]. In addition, the p–n junction-based sensor coated with Ag nanoparticles showed a fast response and recovery time of less than 10 s (Figure 3j), demonstrating a promising sensing performance for real-time detection of CO_2_ gas at low operating temperature of 120 °C.

Recently, the combination of organic polymers and allotropes of carbon (graphene and SWCNTs) was used to reduce the operating temperature down to room temperature for CO_2_ gas detection at a relatively high concentrations (5000 ppm, 2%). Son et al. [52] reported an amine-rich polyethyleneimine (PEI)/graphene organic-inorganic hybrid sensor for fast CO_2_ sensing, demonstrating a high sensing response of 32% compared to 0.4% for PEI-functionalized graphene. A very low bias of 0.1 V was applied during the gas sensing process, resulting in a remarkable “several tens of seconds” for both response and recovery time [52]. Yoon et al. [53] developed a polymer-based chemiresisitve CO_2_ sensor by anchoring a precursor copolymer bearing both 4-vinylpyridine (4VP) groups and azide groups on the surface of SWCNTs resulting in a response of 25% at room temperature for 2% CO_2_ concentration. However, a very long response time of several thousand of seconds was recorded, where full recovery to the baseline never achieved at room temperature. Further studies are required to the sensing performance of organic-inorganic hybrid sensors towards practical CO_2_ gas sensing application [37].

## 3. Health and Medical Monitoring

Breath analysis is an emerging field of medical diagnostics that promises a rapid, real-time, non-invasive, and cost-effective alternative to monitoring techniques such as blood analysis, endoscopy, ultrasonic and tomographic monitoring [54]. VOCs in human breath can reveal a range of common illnesses such as asthma, kidney failure, lung cancer, diabetes, and heart disease. For example, ammonia, isoprene and acetone in the breath can be used to evaluate kidney malfunction, liver fibrosis, and diabetes, respectively [10,55].

### 3.1. Ammonia (NH_3_) 

Ammonia is a corrosive, poisonous and toxic gas with unpleasant smell, which is widely used in various industries including fertilizer, pharmacy and fermentation [56,57]. It may cause burns to the eyes and skin, and respiratory system damage when the concentration is greater than 300 ppm [58]. It is also a promising breath biomarker for detecting and monitoring kidney and liver disease [59,60]. The development of a non-invasive, flexible, reliable, robust and fast-response NH_3_ gas sensor operating at a low temperature is of great interest. However, most commonly used gas sensors are on a rigid substrate with no flexibility, and often operate at elevated temperatures (above 200 °C), hindering their real-world applications as portable miniaturized gas sensors. On the other hand, if flexible substrates such as polydimethylsiloxane (PDMS) [61], cotton fabrics [62], foil [63] and paper [64] can be used, then the gas sensor itself can become lightweight and flexible and can be produced at large scale with the prospect of extensive high-value applications.

**Figure 4:**
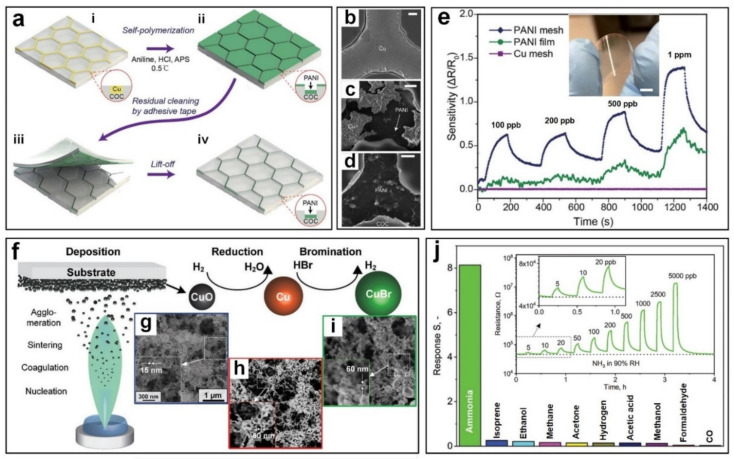
(**a**) The schematic illustrations of PANI mesh preparation. (**a**-**i**) The sacrificial Cu mesh template; (**a**-**ii**) deposition of the hierarchical PANI micromesh on the Cu template via in situ oxidative polymerization of aniline; (**a**-**iii**) removal of the excess residual PANI molecules from the exposed COC by adhesive tape; and (**a**-**iv**) a hexagonal hierarchical PANI micromesh embedded in the COC film. (**b**–**d**) SEM images of the original hexagonal Cu mesh and the PANI mesh after 3 min and 20 min in the aniline polymerization solution, respectively (scale bar is 1 µm). (**e**) The sensing performance of the Cu mesh, PANI film and PANI mesh with different concentrations of ammonia gas ranging from 100 ppb to 1000 ppb. (Inset: the photograph of the transparent PANI mesh ammonia gas sensor). Reproduced with permission from [64,65] Royal Society of Chemistry, 2018. (**f**) Schematic of the CuBr gas sensing film fabricated via flame-aerosol deposition and dry conversion. Inset: SEM images the fractal-like CuO agglomerates after flame-Aerosol Deposition (**g**), lace-like Cu film with more macropores after the dry reduction (**h**) and preserved porous and open morphology of CuBr film after bromination (**i**). (**j**) Sensitivity performance comparison of CuBr films to 500 ppb different gases at 90% RH. Inset: Sensing response of the dry-converted CuBr films to different NH_3_ concentrations at 90% RH. Reproduced with permission from [65,66] Wiley-VCH, 2020.

Recently, electrically conductive nanostructured polymers including polyaniline (PANI) have attracted much attention as potential chemiresistive gas sensors due to their high conductivity and strong response to specific gases. Cai et al. [65] developed a hierarchical nanostructured PANI-based gas sensor (Figure 4a) on a flexible substrate made of cyclic olefin copolymer (COC). The fabrication technique involved the deposition of the hierarchical PANI on a sacrificial Cu micromesh template (Figure 4a(i,ii)) and removal of excess residual (Figure 4a(iii)) resulting in the development of hexagonal PANI micromesh sensor embedded in the flexible COC film (Figure 4a(iv)). The SEM images of Cu mesh, the PANI mesh after 3- and 20-min polymerization are presented in Figure 4b–d. The device featured superior sensitivity towards NH_3_ with a sensing response of 0.7 to 100 ppb at room temperature (Figure 4e) compared to negligible response from PANI film and Cu mesh. This excellent sensing response is attributed to the surface roughness as well as increased specific surface area for the PANI mesh compared to the smoother surface on the PANI film. In addition, the presence of catalytic Cu ions in the PANI mesh resulted in the deposition of emeraldine salt of PANI, leading to higher resistance in the sensors and consequently, a higher response after exposure to NH_3_ gas molecules. The sensing mechanism is based on the chemical absorption of NH_3_ gas molecules on the surface of PANI polymer at room temperature, resulting in lower conductivity of the PANI mesh by a reversible transition from the emeraldine salt to its emeraldine base. 

In another approach, a room-temperature, flexible NH_3_ gas sensor was fabricated by functionalizing the surface of polyethylene terephthalate (PET) fibers with amino group (-NH_2_) followed by PANI coating [67]. A fast response dynamic of 47 s was achieved towards 50 ppm of NH_3_ gas with the sensing response of 1.17. However, poor long-term stability was reported due to degradation of the sensing materials over a week testing period. Similar stability issue was reported for SnO_2_/PANI nanocomposites due to PANI film aging over time [68]. Further investigation in PANI structural modification or seeking alternative polymers may resolve the reduced sensing performance due to polymer degradation.

In addition, the flexible substrate and the embedded PANI mesh (Figure 4e, inset) exhibited acceptable mechanical properties against peeling and bending with less than 15% reduction in the sensing performance after 1000 bending cycles. A low detection limit of 2.5 ppb was observed for this PANI mesh sensor, making this polymer a strong candidate for low concentration detection of NH_3_ gas. However, slow recovery speeds will hinder real-world application as a reliable room temperature gas sensor. 

In another approach, sub-ppb detection of NH_3_ gas molecules (down to 5 ppb) at 90% RH and room temperature with fast response and recovery speed were reported using highly porous (78% porosity) nanostructured CuBr films [66] (Figure 4f). The sensing film was fabricated by continuous deposition of CuO nanoparticles onto Al_2_O_3_ substrates as ultraporous metal oxide film (Figure 4g), followed by dry reduction of CuO to Cu using H_2_ gas (Figure 4h) and then bromination of Cu to CuBr with HB_2_ at 180 °C (Figure 4i). These CuBr films featured an excellent sensing response of 1 towards 10 ppb NH_3_ gas (Figure 4j, inset) and a fast response dynamic of 2.2 min and 50 s for response and recovery time respectively, which is outstanding for room temperature gas sensors at high relative humidity (90% RH). This excellent sensing response is attributed to the unique properties of the fabricated film with 78% porosity and large surface area (Figure 4i,) compared to the conventional CuBr film with a porosity <40%, facilitating rapid gas transport through the ultraporous nanostructure resulting in enhanced resistance modulation. The selectivity of this porous CuBr film was also tested towards other gases including hydrogen, acetone and carbon monoxide, resulting in excellent selectivity towards NH_3_ with a selectivity of higher than 30 which is superior to most state-of-the-art NH_3_ gas sensors. (Figure 4j). 

### 3.2. Isoprene (C_5_H_8_) 

Isoprene is a breath biomarker that could be used to quickly screen individuals for high blood cholesterol [69]. Healthy adults exhale a concentration of 22 to 234 ppb isoprene [70,71], however, patients with high cholesterol exhale much less. It is reported that the concentration of isoprene in human breath decreases by ~35% when patients are treated with cholesterol-lowering lova and atorvastatins [69]. Similar changes were reported in the breath composition of patients with lung cancer [71,72] and liver disease [73], indicating the importance of isoprene sensors in medical applications. The challenge, however, is to find a suitable material capable of detecting low concentration of isoprene sufficiently at high relative humidity and low operating temperature without any interference from other breath compounds. 

Using flame spray pyrolysis, Guntner et al. [70] presented an isoprene selective chemiresistive sensor made of Ti-doped ZnO nanoparticles (with an average particle size of 20 nm) forming an ultraporous nanostructured film. A superior isoprene response of 0.82 towards 500 ppb was observed at an optimal Ti content of 2.5 mol% at 90% RH and an operating temperature of 325 °C. The excellent sensing performance at such a high relative humidity is attributed to substantial incorporation of Ti^4+^ cations into the ZnO wurtzite lattice, creating point detects in the crystal and impeding nanoparticle growth during flame synthesis. In addition, Ti^4+^ sites dissociate water and chemisorb the resulting hydroxyl groups that interact with isoprene, enhance the sensitivity and selectivity of Ti-doped ZnO isoprene gas sensors at 90% RH compared to pure ZnO nanostructured sensors.

Very recently, Noriko et al. [74] reported a highly sensitive isoprene gas sensor using pyramid shaped ZnO particles loaded by Au nanoparticles (3 nm in diameter) and synthesized via solvothermal method followed by 4 h annealing at 425 °C. The fabricated sensing material demonstrated a sensing response of 0.79 towards low concentrations of isoprene (50 ppb) under 80% RH at 350 °C. Enhanced selectivity compared to acetone, ethanol and hydrogen was confirmed in Au-doped samples with an estimated detection limit of 6 ppb, which meets the requirements for diagnosis of liver disease and sleeping state by breath analysis [69]. However, its high operating temperature of 350 °C currently required would restrict the commercial viability of this approach. 

In order to reduce the operating temperature, Han et al. [75] developed a flower-like In_2_O_3_ nanostructure using a simple hydrothermal technique, to detect isoprene gas molecules with a very low concentration (5 ppb) at 190 °C. The nanostructure exhibited excellent long-term stability and a quick response dynamic with response time of 53 s. This relatively low operating temperature (190 °C) extends the working life and facilitates the portable application of the sensor. However, further studies are required to investigate the sensor’s selectivity towards isoprene upon exposure to a complex gas mixture as achieving a high selectivity towards the targeted gas is still one of the major challenges restricting the applications of metal oxide-based gas sensors. This challenge is more significant regarding isoprene detection due to the absence of distinct functional groups that can be exploited for selective sensor interaction.

**Figure 5:**
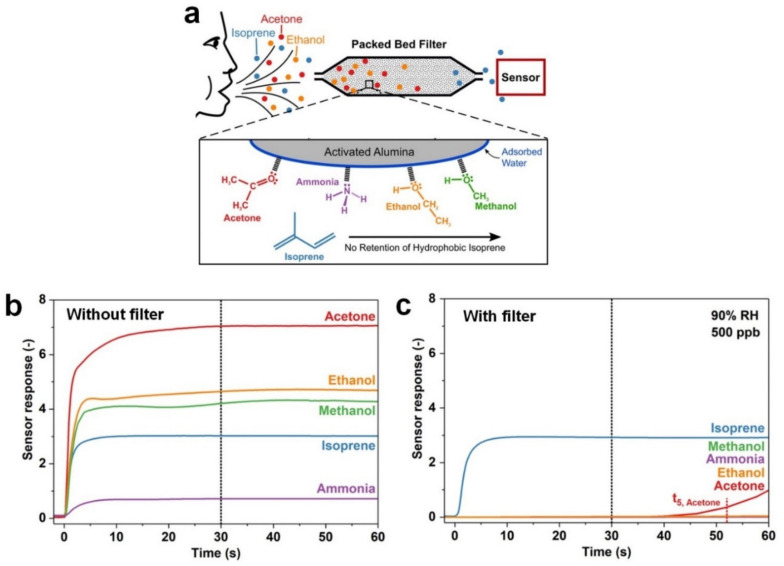
(**a**) Filter-sensing concept for selective isoprene detection in complex gas mixtures. Responses of a Pt-doped SnO_2_ sensor at 90% RH to 500 ppb of isoprene and other gases (**b**) without and (**c**) with activated alumina filter. Using the activated alumina filter, hydrophilic analytes are held back until their characteristic breakthrough time t_5_, while the response of isoprene is unchanged. Reproduced with permission from [76] American Chemical Society, 2018.

An activated alumina filter with a high surface area adsorbent was used to enhance the selectivity of Pt-doped SnO_2_ sensors towards hydrophobic isoprene gas by adsorbing and retaining hydrophilic gaseous molecules in a gas mixture (Figure 5a) [76]. Using this technology, the hydrophilic gases including acetone, ethanol, methanol and ammonia were retained by the activated alumina filter while isoprene molecules with hydrophobic properties were passed unhindered. This innovative approach resulted in outstanding selectivity of >100 towards low concentration isoprene (down to 5 ppb) in a complex gas mixture at 90% RH (Figure 5b,c). The filter-based sensing technology featured great stability in sensing performance even after 8 days of continuous gas exposure. However, this Pt-doped SnO_2_ sensor needed to be maintained at 400 °C by a substrate back-heater during the whole process, a requirement that will result in high power consumption and shorter sensor lifetime. Meanwhile, the activated alumina filter may not work desirably for selective detection of isoprene in the presence of other hydrophobic gases in exhaled breath.

### 3.3. Acetone (C_3_H_6_O) 

Acetone concentrations in exhaled breath could be potentially indicated as a novel biomarker for non-blood based diabetic diagnostics and monitoring, specifically for Type I diabetes [77]. This is because a healthy individual exhales 300–900 ppb acetone gas, but the concentration can easily exceed 1800 ppb for diabetes mellitus patients [78,79]. It was assessed that diabetes directly caused 4% of premature (under age 70) mortality from non-communicable diseases (NCDs) [80], and the death toll is predicted to continue to double until 2030. The prevalence of diabetes mellitus is a worldwide issue, and the development of user-friendly portable non-invasive breath gas sensors would make a huge difference to patient outcomes. New materials and technologies that can be applied to detect the abnormality of acetone concentrations at an early stage of diabetes development, and the novel design of breath gas sensors that could offer a user-friendly monitoring features, such as portable non-invasive technologies, can greatly contribute to save more lives.

Despite significant progress in developing acetone sensing technologies, the sensitivity and detection limits of metal oxide semiconductor gas sensors at low temperatures are only in the range of sub-ppm [81,82]. Further improvements are required to enhance their sensitivity to ppb as well as high selectivity towards the targeted gas, particularly at low temperature and high relative humidity. A few materials have met the demands of ppb level detection for healthcare, but they were mostly decorated with noble metals [82] or incorporated minor metals [83,84,85,86], which is ideal for practical sensing devices.

Lu et al. [83] reported a fast responding (5 s response/recovery time) acetone gas sensor based on monoclinic and hexagonal phase of WO_3_ nanocrystalline fabricated by one-pot microwave assisted hydrothermal method. Increasing the operating temperature up to 320 °C resulted in an outstanding sensitivity in ppb-level with a LOD of 7.5 ppb. This sensing performance could be attributed to the oxygen vacancies, different polarities from WO_3_ nanocrystalline geometry and the strong affinity between monoclinic WO_3_ and acetone molecules. However, its real-world application could be limited due to high operating temperature.

**Figure 6:**
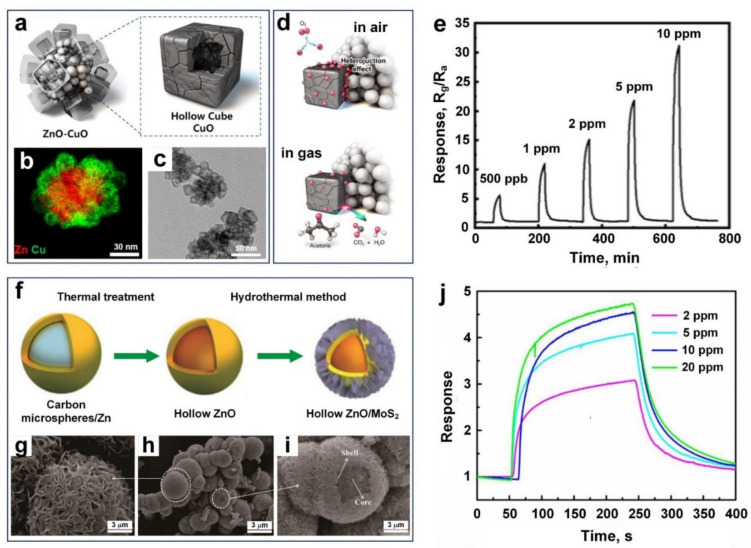
(**a**) Schematic, (**b**) elemental mapping, and (**c**) TEM image of the ZnO-CuO core-hollow cube nanostructures. (**d**) Schematic illustration of gas sensing mechanism upon exposure to acetone gas molecules. (**e**) Time-transient response curves of the sensors with a dynamic range of acetone concentrations at 200 °C. Reproduced with permission from [46] American Chemical Society, 2020. (**f**) The schematic fabrication diagram, and (**g**–**i**) SEM images of HZnO/MoS_2_ core/shell heterogeneous structures. (**j**) The UV-assisted response dynamic of HZnO/MoS_2_ to 2–20 ppm acetone gases. Reproduced with permission from [84] Elsevier, 2020.

Lee et al. [46] developed a novel p–n heterojunction chemiresistive sensor made of p-type CuO hollow nanocubes with an edge size of 15 nm attached to the surface of n-type ZnO spherical cores of 50 nm in diameter (Figure 6a,b). The fabricated ZnO-CuO core-hollow cube nanostructures presented a high sensing response of 10.14 towards 1 ppm acetone concentration at 200 °C with a LOD of 9 ppb. This superior sensing response could be attributed to the unique morphology of these core-hollow nanostructures with excellent gas penetration through the active material, their small grain size (7 and 4 nm for ZnO and CuO, respectively) and large surface area (336 m^2^ g^−1^). 

The sensing mechanism is based on resistance modification through controlling the potential energy barrier at the interface and the narrowing of the conduction channel in fabricated p–n junctions within the core-hollow nanostructure. Before treatment charge depletion layers were created at the p–n junction interface, increasing the electrical resistance of the sensing materials. However, upon exposure to the acetone molecules, surface-adsorbed oxygen molecules react with the acetone, pushing the depletion layers into the CuO domains. The resistance eventually increases through the interparticle p−p junctions, thus leading to such a high sensitivity and a low detection limit. However, there is still an increasing need to explore promising detection methods with the potential to provide outstanding acetone sensing at lower operating temperatures. 

Using a hydrothermal method, Chang et al. [84] developed a core-shell heterogenous structure made of hollow ZnO spheres with 40 nm wall thickness, and decorated by 2D MoS_2_ nanosheets grown perpendicularly on the ZnO surface (Figure 6f–i). These nanoflower-shaped MoS_2_ nanosheets played a key role in protecting the hollow ZnO spheres from aggregation while accelerating gas diffusion through sensing materials, resulting in a fast response/recovery speed (56 s/69 s) (Figure 6j). The sensing mechanism is based on the formation of nanostructured p–n heterojunctions between hollow ZnO spheres and 2D MoS_2_ nanosheets, resulting in an electrostatic field formation at their interface, enhancing the sensing performance of the fabricated device compared to the pure counterparts. Here, ultraviolet (UV) light radiation was introduced to further improve acetone response and to significantly reduce the working temperature (down to 30 °C). A sensing response of 0.34 towards 100 ppb acetone was achieved at 100 °C, under UV illumination. This excellent sensing response towards acetone is the highest reported so far at such a low operating temperature and is attributed to the synergistic effect of enhanced UV light harvesting (due to the decoration of hierarchical MoS_2_ nanosheets on the surface of hollow ZnO spheres) and the unique morphology of the hollow ZnO/MoS_2_ p–n heterojunctions nanostructure. 

## 4. Summary and Outlook

In conclusion, we reviewed some recent achievements for the development of small, non-invasive, portable, and flexible sensor technologies for highly sensitive, selective measurement of targeted gases for air quality, environmental monitoring, health, and medical applications. Amongst a wide variety of organic and inorganic nanomaterials, metal oxide nanostructured sensors demonstrated the highest sensitivity and selectivity with the lowest LOD. For instance, In_2_O_3_ nanostructured sensors demonstrated high sensitivity and selectivity towards targeted gases. The addition of extraneous metal, such as Au, Pt and Ti via doping or coating significantly improved the sensing performance, however, a relatively high operating temperature (150–400 °C) is still required to activate the sensing material, which hinders their real-world application.

The introduction of nanostructured materials including rGO and graphene, as well as the utilization of other external activation sources including UV and visible light, has dramatically reduced the required operating temperature to low (100 °C) or room temperature (20–30 °C), resulting in a significant energy consumption reduction. This sheds a light on new approaches for nanostructured gas sensor designs via taking advantage of additional energy source, such as light illuminations and/or electromotive forces (voltages) rather than thermal energy to trigger and/or maintain the sensing actuation.

Design, fabrication and sensing mechanism of hybrid films, nanocomposites, doped nanomaterials, nanoscale p–n heterojunctions, yolk-shell nanostructures, flexible polymer-based sensors and ultraporous nanofilms were discussed in detail. Despite significant advancements in this area, the major standing challenges include slow response dynamics and lack of selectivity towards targeted gas are still unresolved. New technologies with innovative nanoscale designs could be employed to provide sufficient filtering function to block irrelevant gases and consequently, enhance the sensor’s selectivity without compromising its sensitivity.

Nonetheless, optimization of the sensing materials and mechanism for room temperature operating is already becoming an enabling tool for the utilization of nanostructured gas sensors in real-world applications. Materials with porous, ultraporous and hollow sensing nanostructures provides a meaningful and promising avenue to improve the gas sensing performance due to the increased specific surface area and surface to volume ratio. The future development of low temperature sensing technologies is bright, with applications in many aspects of technology, industry, or daily life, providing strong social and financial benefits for addressing the large number of standing fundamental and technological challenges.

**Table 1:** The key figures of merit of state-of-the-art nanostructured gas sensors.

Target Gas	Materials	Methods	Working Temp. (°C)	Con. (ppm)	Response ^#^	Response/Recovery Time	Ref.
NO_2_	SnS_2_/rGO	Hydrothermal	RT	1	6.5 ^a^	75 s/~180 s *	[18]
	rGO/ZnO_1−x_	Hydrothermal	25	0.1	4.66 ^b^	1.5 min/2.5 min	[26]
	ZnO/rGO	Solvothermal	110	2.5	32.11	182 s/234 s	[29]
	SnS_2_/rGO	Wet chemistry	80	10	0.618 ^c^	6 min/~53 min	[30]
	SnS_2_	Wet chemistry	120	10	~35	~170 s/~140 s	[31]
	ZnO/rGO	Thermal reduction, soft solution	RT	5	2.5	25 s/15 s	[87]
	g-C_3_N_4_/rGO	Layer-by-layer self-assembly	RT	2	0.52	138 s/318 s	[88]
	Zn_2_SnO_4_/rGO	Hydrothermal	30	1	0.83	-	[89]
	S-rGO/SnS_2_	Hydrothermal	RT	1	0.72	-	[90]
	CdS/ZnO	Liquid plasma spray	RT	1	30.9 ^d^	18.2 min/>70 min	[91]
	In/Ga/ZnO	RF sputtering	RT	5	0.5 ^e^	-/5 min ^e^	[92]
	SnO/SnO_2_	Hydrothermal	RT	0.2	1.5	57 s/5 min	[93]
	Si/WO_3_	Chemical etching & annealing	RT	5	0.92	1 s/31 s	[94]
	NiO/CuO	Reflux, hydrothermal	RT	100	0.772	2 s/-	[95]
	α-Fe_2_O_3_/PANI	Polymerization	RT	20	228	2.3 min/2.4 min	[96]
	CuPcTS/SnO_2_	Spin coating	50	1	2399	5 min/10 min	[97]
SO_2_	Ni_3_BTC_2_/OH-SWNTs	Solvothermal	RT	15	0.85 *	5.59 s/11.04 s	[33]
	Cu:SnO_2_	Precipitation	250	6	90.51	4.5 min/15 min	[34]
	g-C_3_N_4_/rGO	Layer-by-layer self-assembly	RT	20	0.09 ^f^	140 s ^f^/130 s ^f^	[88]
	rGO/WO_3_	Metal organic decomposition	25	0.3	0.027	66 s/298 s	[98]
	Au/SnO_2−X_	Hydrothermal	200	20	0.904	34 s/14 s	[99]
	PANI	Template-free	RT	5	0.045	185 s/<200 s	[100]
	Ru/Al_2_O_3_/ZnO	Hydrothermal & inkjet printing	350	25	0.2	~1 min/~6 min	[101]
	NH_4_^+^ZSM-5 (23)	Ion exchange	RT	4200	0.85	63 min/3 min	[102]
CO_2_	CeO_2_	Solvothermal	100	2400	2.9	2.58 min/4.08 min	[48]
	Ag/CuO/BaTiO_3_	Hydrothermal	120	700	0.4	3 s/8 s	[49]
	Graphene PEI/PEG	CVD & e-beam evaporation	RT	5000	0.3 ^g^	“several tens of seconds”	[52]
	SWCNTs/Q4VP−VBAm	Spray coating	21	20000	0.25 ^*^	<1000 s */~3000 s	[53]
	Pd/La_2_O_3_	Spray pyrolysis	250	500	2.57	105 s/145 s	[103]
NH_3_	PANI	Polymerization	24	0.0025	0.03	-	[65]
	CuBr	FSP	RT	5	276	2.2 min/50 s	[66]
	PET/MWCNTs/PANI	EDA modification	~15–18	50	1.17	47 s/-	[67]
	SnO_2_/PANI	Hydrothermal, polymerization	28	100	28.8	125 s/167 s	[68]
	S-rGO/SnS_2_	Hydrothermal	RT	20	0.45	-	[90]
	SWCNTs	Hydrothermal	RT	1.5	0.032	10 min/- ^h^	[104]
C_5_H_8_	Ti:ZnO	FSP	325	0.02	0.26	~1 min/~5 min	[70]
	Au/ZnO	Solvothermal, annealing	350	1	~1	~2 min */-	[74]
	In_2_O_3_	Hydrothermal	190	5	0.75	53 s/299 s	[75]
	Pt:SnO_2_	FSP	400	0.5	~0.75	10 s/20 s *	[76]
	Pt/In_2_O_3_	Hydrothermal	200	5	0.99	124 s/204 s	[105]
	Cr_2_O_3_:In_2_O_3_	Hydrothermal	240	0.5	0.95	135 s/830 s	[106]
C_3_H_6_O	ZnO/CuO	Thermal oxidation	200	1	10.14	-	[46]
	In/Ga/ZnO	Photochemical activation	RT	750	0.27 ^i^	37 s/53 s	[81]
	Au/ZnO	Precipitation	172	100	0.98	1 s/20 s	[82]
	WO_3_	Hydrothermal	320	0.25	0.97	5 s/5 s	[83]
	HZnO/MoS_2_	Hydrothermal	30	50	~0.25 ^j^	19 s/97 s	[84]
	Co_3_O_4_/SnO_2_	Hydrothermal	220	50	0.92	12 s/18 s	[85]
	Co_3_O_4_/CoWO_4_	Hydrothermal	255	20	8.9	22 s/12 s	[86]
	Au/ZnO	Pyrolysis, sonication	365	100	~1	5 s/-	[107]

**^#^** The mathematic expression of sensing response (S) varies in some literatures, herein, it is converted correspondingly via the unified formula of $S=\frac{\left|R_{g}-R_{a}\right|}{R_{a}}$, or $S=\frac{\left|I_{0}-I\right|}{I}$, where $I_{0}$ and I are the currents detected before and after targeted gas exposure respectively. * Estimated/calculated from the graph, data is unavailable; ^a^ Assisted under Red light (650 nm, 1 mW·cm^−2^) and bias (5–15 V); ^b^ Assisted under white-light illumination with a light density of 0.15 W·cm^−2^; ^c^ Assisted by applying a bias of 1 V; ^d^ Assisted under Green light (510 nm, 0.05 W·cm^−2^); ^e^ Assisted under blue light (450 nm, 1 mW·cm^−2^); ^f^ Assisted under UV light illumination and the bias of 3 V; ^g^ Assisted by applying a bias of 0.1 V; ^h^ A local heater or a UV LED has to be used for the full recovery of the sensor after each sensing cycle; ^i^ Assisted under UV light (390 nm, 30 mW·cm^−2^); ^j^ Assisted under UV light (375 nm, 50 μW·cm^−2^).

## Data Availability

Not applicable.
